# Structural change in feedlot cattle death loss rates

**DOI:** 10.3389/fvets.2023.1087080

**Published:** 2023-01-30

**Authors:** Mark Buda, Kellie Curry Raper, John Michael Riley, Derrell S. Peel

**Affiliations:** ^1^Department of Agribusiness and Bioresource Economics, Faculty of Agriculture, Universiti Putra Malaysia, Serdang, Selangor, Malaysia; ^2^Department of Agricultural Economics, Oklahoma State University, Stillwater, OK, United States

**Keywords:** cattle, feedlot, mortality, parameter instability, profitability

## Abstract

**Introduction:**

Industry reports and anecdotal evidence indicate that the death loss rate in cattle feedlots has increased over time. Such increases in death loss rates impact feedlot cost and thus profitability.

**Objectives:**

The primary objective of this study is to examine whether feedlot death loss rates in cattle have changed over time, to analyze the nature of any identified structural change, and to identify possible catalysts for that change.

**Methods:**

Data from the Kansas Feedlot Performance and Feed Cost Summary from 1992 through 2017 is used to model feedlot death loss rate as a function of feeder cattle placement weight, days on feed, time, and seasonality in the form of monthly dummy variables. Commonly used tests of structural change, including the CUSUM, CUSUMSQ, and Bai and Perron methods, are implemented to examine the existence and nature of any structural changes in the proposed model. All tests indicate the presence of structural breaks in the model, including both systematic change and abrupt change. Following a synthesis of structural test results, the final model is modified to include a structural shift parameter for the period from December 2000 to September 2010.

**Results:**

Models indicate that days on feed has a significant positive influence on death loss rate. Trend variables indicate that death loss rates have increased systematically over the period studied. However, the structural shift parameter in the modified model is positive and significant for December 2000 to September 2010, indicating that death loss is higher on average during this period. Variance of death loss percentage is also higher during this period. Parallels between evidence of structural change and possible industry and environmental catalysts are also discussed.

**Conclusions:**

Statistical evidence does indicate changes in the structure of death loss rates. Ongoing factors such as changes in feeding rations prompted by market forces and feeding technologies may have contributed to systematic change. Other events, such as weather events and beta agonist use could result in abrupt changes. No clear evidence directly connects these factors to death loss rates and disaggregated data would be required to facilitate such a study.

## Introduction

Some degree of death loss (mortality) is unavoidable in beef cattle feedlot operations. That is, the number of fed cattle sold will be less than the number of feeder calves purchased because a small percentage of animals will die during the feeding phase. However, while productivity has increased in the beef industry as indicated, for example, by beef carcass weights increasing an average of 2.27 kilos a year for the last 25 years, feedlot death loss rates have also been increasing over time (1). United States Department of Agriculture, National Health Monitoring System (USDA-NAHMS) reports average feedlot death rates of 1.4% for small feedlots (1,000–7,999 head capacity) and 1.6% for large feedlots (>8,000 head capacity) for 2011, representing increases of 36% and 23%, respectively, over 1999 measures (2). During the twenty-five year period from January 1992 to July 2017, the mean monthly death loss percentage for steers more than doubled from 0.70% to 1.74% in feedlots represented by the Kansas Feedlot Performance and Feed Cost Summary (3). Data from Elanco Animal Health and the Benchmark^®^ Performance Program indicated that death loss in associated feedlots was flat from 2005 through 2010 and then began increasing after 2010 (4). They report that from January 2005 to September 2014, steer death loss increased from 1.34 to 1.71% and heifer death loss increased from 1.41 to 1.84%, with much of that increase coming after 2010. Maday (5) reports similar results from Professional Cattle Consultants (PCC) data which indicates that death loss doubled from 2010 to 2015.

Feedlot death loss rates impact economic profits. Amount of saleable product, feed conversions, average daily gain, and cost of gain are all affected. Irsik et al. (6) reported that a one% increase in death loss per pen resulted in a 0.122 kg increase in feed to gain ratio and a 0.036 kg decrease in average daily gain, indicating that more feed is needed to gain the same amount of weight on average and increasing feedlot cost by approximately $1 per head. Death loss impacts profitability through increased costs per live animal kilograms sold for costs such as feed, medical treatment, labor, manure disposal, and animal disposal (7). Roeber et al. (8) found that economic losses from death loss are highly correlated with morbidity (sickness). In their study, death loss per pen increased by 0.14% for a one precent increase in the number of medical treatments, in turn impacting the average cost invested in head sold.

Several studies have evaluated feedlot death loss over time. Babcock et al. (9) found significant increases in feedlot death loss from 1992 to 2004, with seasonal trends in both steer and heifer death loss. Using private data from feedlot veterinary consultants, Loneragan et al. (7) examined yearly death loss relative to specific causes of death (BRD, digestive disorders, and other disorders) and found that death loss increased 38% from 1994 to 1999. Engler et al. (10) analyzed private feedlot data (2001–2013) and concluded that feedlot death loss for three placement weight classes of steers (272.2, 317.5, and 362.9 kg) exhibited an increasing trend.

Structural change can be defined as shifts or evolutions in market or industry functions and is evidenced in parameter instability. The change can be gradual or abrupt and permanent or temporary. If death loss rate in feedlot cattle is changing over time, it is important to understand the nature of the change. Changes in death loss may be attributed to specific events such as extreme weather or disease outbreaks which have an immediate impact. Alternatively, changes in death loss rates may be the indirect or delayed impact of policy change, technological advancement in animal health management or efficiencies, or even changes in management or sourcing. For example, Belasco et al. (11) found that various pen characteristics influence mortality. Factors that impact death loss can be categorized into controllable and uncontrollable factors. Controllable factors include technology adoption, feed rations, and cattle sourcing. Feedlot decisions regarding technology adoption may depend on immediate costs and returns projections, but long-term implications may be less clear. The complexity of the beef cattle industry adds to the challenge of managing disease (and death loss) in feedlots because disease management likely extends beyond the feedlot into other production sectors (1).

In the present analysis, we examine whether feedlot death loss has changed over time, analyze the nature of any structural change, and identify possible sources of change. This study differs from Babcock et al. (9) by considering days on feed in addition to placement weight and seasonality as well as assessing what type of structural change may have occurred. It also differs from other investigations by incorporating a longer period of time and considering more recent data periods. Cumulative sum tests (CUSUM), CUSUM of squares (CUSUMSQ) and Bai and Perron testing procedures are used to assess whether structural change exists in feedlot death loss rates and to determine whether the structural change is systematic, abrupt or both.

## Materials and methods

Data from the Kansas Feedlot Performance and Feed Cost Summary is used for this study and was obtained from the Livestock Marketing Information Center (LMIC). The data contains feedlot performance and closeout data from a monthly survey of Kansas commercial cattle feeding operations. We focused on steer data from January 1992 to July 2017 since death loss percentage is not available in prior reports. Data are reported by closeout month and include death loss percentage, in-weight, and average days on feed and are summarized in Table 1. Data are the means of individual feedyard monthly averages.

**Table 1:** Monthly death loss percentage, placement weight^*^, and average days on feed for steers, Kansas feedlot summary, January 1992–July 2017.

**Variables**	**Unit**	**Mean**	**Standard deviation**	**Minimum**	**Maximum**
Death loss percentage	%	1.22	0.49	0.35	2.78
Placement weight	kg.	355.9	18.5	309.0	397.6
Average days on feed	days	151	12	119	186

Source: Livestock marketing information center (3).

* Weight unit originally reported in pounds.

Structurally, a basic model of death loss in feedlot production can be written as


(1) $$\begin{array}{l} \text{Ln}(DL_{t})=\beta_{0}+\beta_{1}Ln(INWT_{t})+\beta_{2}\text{Ln}\left(DOF_{t}\right)\\ \;+\beta_{3}t+\sum_{k=1}^{11}\delta_{k}MD_{kt}+\varepsilon_{t} \end{array}$$

where *t* = 1, …, *T* denotes as closeout month, Ln(*DL*_*t*_) is the natural log of death loss percentage, Ln(*INWT*_*t*_) is the natural log of in-weight, i.e., placement weight, *Ln*(*DOF*_*t*_) is the natural log of days on feed, *t* is time trend, *MD*_*kt*_ are monthly dummy variables from October to August, ε_*t*_ is the error term, and $\varepsilon_{t}\sim N\left(0,\sigma^{2}\right)$.

**Figure 1:**
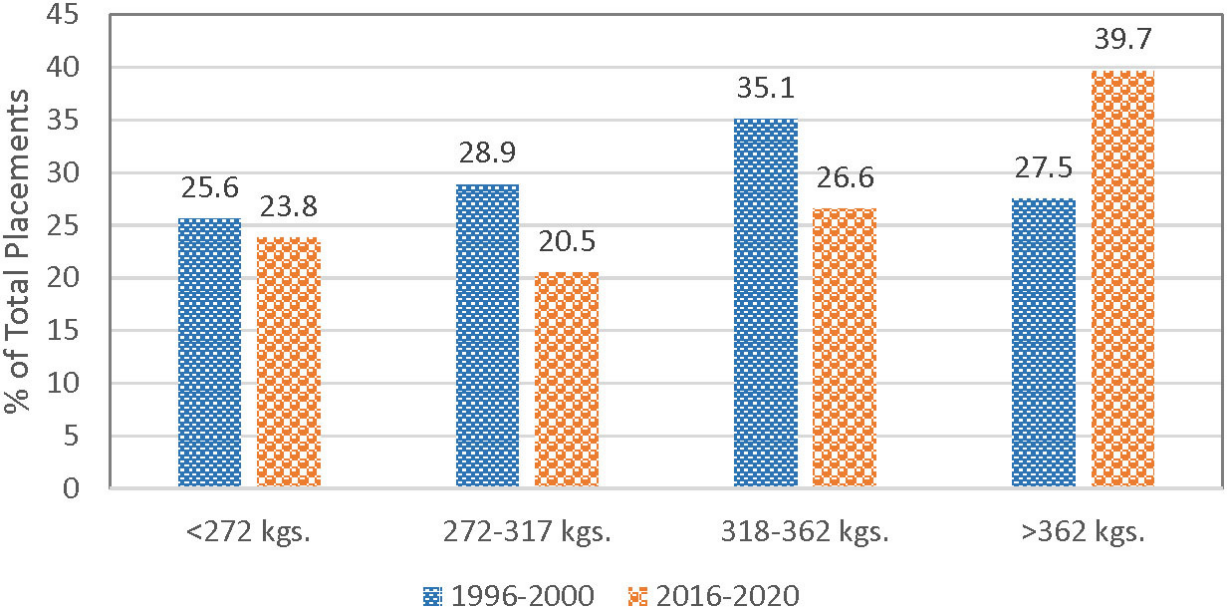
Comparison of feedlot placement distributions by placement weight (kilograms), 1996–2000 and 2016–2020. Source: USDA-NASS Monthly Cattle on Feed Reports, Data compiled by LMIC.

**Figure 2:**
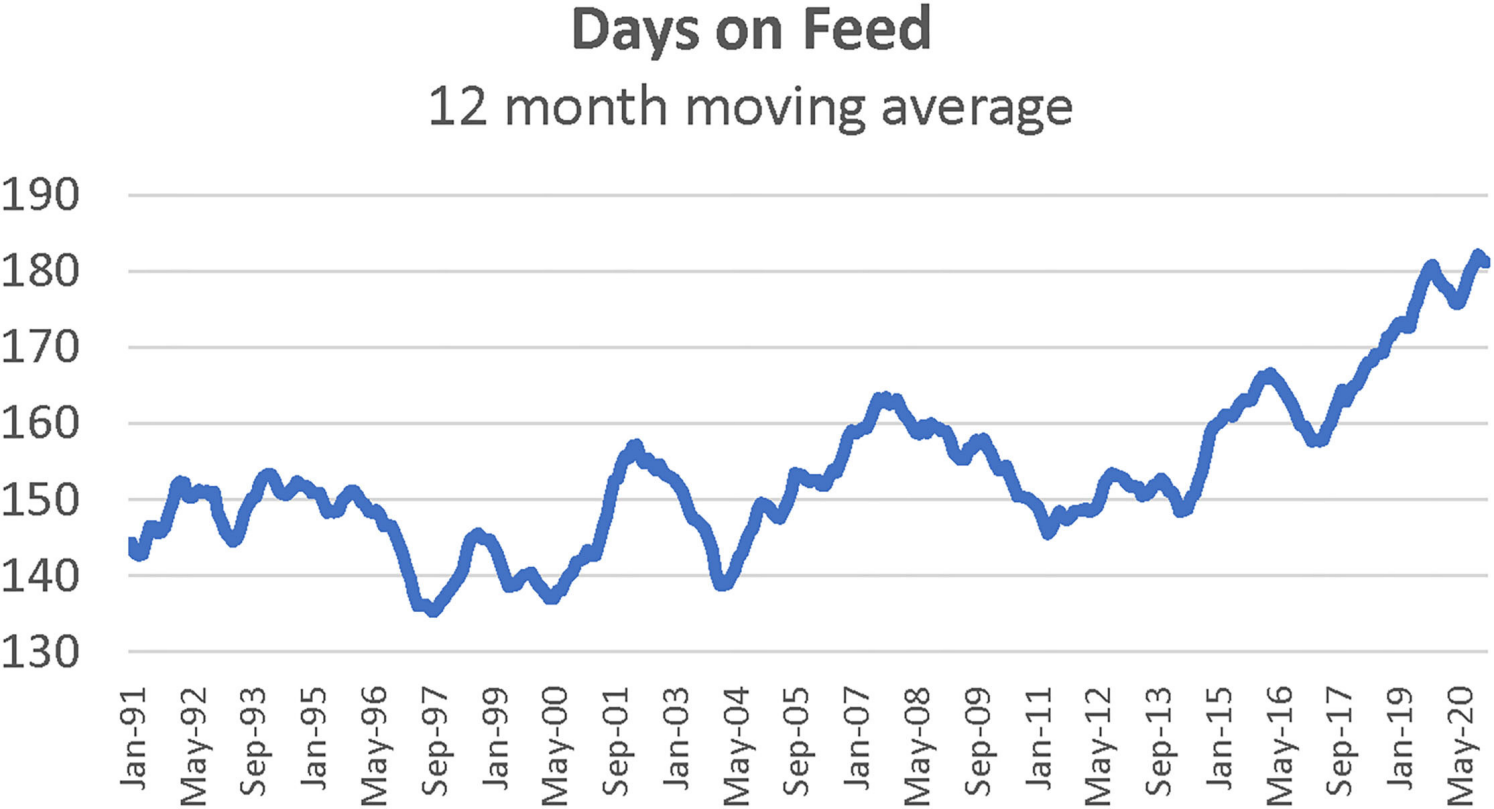
Twelve month moving average of days on feed for fed cattle, January 1991–May 2020. Source: Kansas Feedlot Performance and Feed Cost Summary, Data compiled by LMIC.

In-weight is included since previous research suggests that placement weight may influence death loss. Light weight feeder calves may be more prone to sickness and stress, increasing the possibility of death relative to heavier feeder calves during the early stage of feeding period. In particular, lighter weight feeders are more susceptible to respiratory illness (7). Intuitively, lighter placement weights would also lead to longer days on feed. Pen-level data from feedlots confirms that lightweight placements require additional days on feed (12). However, most recent research uses aggregate data (i.e., average placement weights) where the correlation between placement weight and days on feed is less strong (13, 14). For example, average aggregate placement weights do not fully reflect changes in the distribution of feedlot placements by weight (Figure 1). Market signals such as high fed cattle prices or low feeder supply may prompt feedlots to feed cattle longer. Advancements in cattle feeding technologies can increase the point of diminishing returns to weight gain for an individual animal, creating economic incentives for longer days on feed. Average days on feed have increased more sharply in recent years (Figure 2). Even with this economic incentive, it is possible that increasing days on feed may lead to higher death rates. Thus, days on feed is included in the model. Monthly dummy variables are included in the model to capture any seasonal patterns in feedlot death loss rates that may exist because of environmental factors such as temperature, relative humidity, snow, wind, rain, and mud.

The model in Equation 1 was estimated using SAS 6.1. Variations of the model were first estimated and compared using AIC and SBC tests for model fit. For brevity, only results from Equation 1 as presented here are reported. After testing for misspecifications, including normality (Jarque-Bera and omnibus test), functional form (Kolmogorov-Gabor polynomial and Ramsey RESET tests), and autocorrelation, the model was corrected for first order autocorrelation using the PROC AUTOREG procedure.

### Measuring structural change

Structural change can be examined using a variety of statistical methods. Chow (15) test is commonly used to determine the existence of abrupt change when there is one known potential breakpoint, dividing the sample into two sub-samples at the suspected breakpoint. The null hypothesis presumes no structural change; that is, model parameters are stable over the full sample. The alternative hypothesis is structural change at the suspected breakpoint. The CUSUM, CUSUMSQ, and Bai and Perron (16) testing procedures are extensions of the Chow test.

The CUSUM test analyzes recursive residuals to detect systematic change in model parameters when the breakpoints are not known. The sum of the recursive residuals is plotted and compared to a critical bound. The test is based on the plot of the following quantities:


(2) $$\begin{array}{l} W_{t}=\overset{m}{\underset{t=n+1}{\sum }}\frac{w_{t}}{{\hat{\sigma }}_{w}} \end{array}$$

where,


(3) $$\begin{array}{l} {\hat{\sigma }}_{w}^{2}=\frac{\sum_{t=n+1}^{m}{\left(w_{t}-\bar{w}\right)}^{2}}{T-n-1},\;\bar{w}=\frac{\sum_{t=n+1}^{m}w_{t}}{T-n},\;m=n+1,\;\ldots ,\;T \end{array}$$

and where *W*_*t*_ is the sum of the recursive residuals, *w*_*t*_ is the recursive residual, ${\hat{\sigma }}_{w}$ is standard error of the recursive residual, *m* is the unknown breakpoint, *n* is the minimum sample size required for model fit, and *T* is total sample size. The critical bound is given as


(4) $$\begin{array}{l} B_{W}=\frac{a\left(2m+T-3n\right)}{\sqrt{T-n}} \end{array}$$

where *a* is equal to 0.948 for significance at the 5% level. The null hypothesis of no structural change is rejected if *W*_*t*_ crosses the boundary of [−*B*_*W*_, *B*_*W*_] (17). An important point to note is that the CUSUM test only detects instability of the intercept (18, 19).

Brown et al. (20) suggest the CUSUMSQ test to determine whether structural change is random or abrupt, detecting variance instability. The CUSUMSQ test is similar to the CUSUM test, but uses the cumulative sum of squared recursive residuals, computed as


(5) $$\begin{array}{l} S_{t}=\frac{\sum_{t=n+1}^{m}w_{t}^{2}}{\sum_{t=n+1}^{T}w_{t}^{2}} \end{array}$$

where *S*_*t*_ is the cumulative sum of squared recursive residuals and *m, n*, and *T* are previously defined in equation 2. The critical bound is calculated as


(6) $$\begin{array}{l} B_{S}=c+\left(\frac{m-n}{T-n}\right) \end{array}$$

where *c* is equal to 1.358 for significance at the 5% level. The CUSUMSQ test has poor asymptotic power because the possibility of rejecting the false null hypothesis (H_0_: no structural change) becomes lower as the number of observations move toward infinity which means a greater chance of type II error (21, 22). However, this is not an issue in this study because the number of observations is relatively small.

An alternative approach when the number of breakpoints is unknown is to use the tests proposed by Bai and Perron (16) and Bai and Perron (23). Bai and Perron propose three structural change tests to find the number and location of the breakpoints simultaneously. These tests use the sup-F statistic which is the maximum F-statistic of the Chow test. The null hypothesis of no breakpoints is tested against the alternative of *m* known number of breakpoints. The sup-F statistic is


(7) $$\begin{array}{l} \begin{array}{lll} \text{supF}({\text{λ}}_{1},\ldots ,\;{\text{λ}}_{m},q) & = & \frac{T-(m+1)q-p}{mq} \end{array}\\ \;\begin{array}{ll} \times & \frac{R^{{}^{\prime }}{\hat{\beta }}^{{}^{\prime }}{\left[RI{\left(\hat{\beta }\right)}^{-1}R^{{}^{\prime }}\right]}^{-1}R\hat{\beta }}{SSR_{m}} \end{array} \end{array}$$

where *T* is total sample size, *q* is number of restrictions, *p* is number of explanatory variables, *R* is a conventional matrix such that $R^{{}^{\prime }}{\hat{\beta }}^{{}^{\prime }}=\left(\beta_{1}^{{}^{\prime }}-\beta_{2}^{{}^{\prime }},\ldots ,\beta_{m}^{{}^{\prime }}-\beta_{m+1}^{{}^{\prime }}\right)$, *I* is the identity matrix, and *SSR*_*m*_ is the sum of squared residuals under the alternative hypothesis.

To improve the robustness of this test, Bai and Perron (23) use a double maximum F-statistic given an upper bound and weighted upper bound. Fixing an upper bound for m, the double maximum F statistic is given below:


(8) $$\text{DmaxF}\left(M,\;d_{1},\ldots ,\;d_{M},q\right)=\underset{1<m<M}{\max}d_{m}\text{supF}\left({\text{λ}}_{1},\ldots ,\;{\text{λ}}_{m},q\right)$$

where *M* is the chosen upper bound for number of breaks and (*d*_1_, …, *d*_*M*_) are fixed weights that reflect some information about the chosen upper bound. In the weighted double maximum F statistic, *d*_1_ = 1 and *d*_*m*_ = *c*(*q*, α, *m*) for *m*>1. The weighted double maximum F statistic can be written as:


(9) $$\text{WDmaxF}\left(M,q\right)=\underset{1<m<M}{\max}\frac{c(q,\alpha ,1)}{c(q,\alpha ,m)}\text{supF}\left({\text{λ}}_{1},\ldots ,\;{\text{λ}}_{m},q\right)$$

where *c*(*q*, α, *m*) is the asymptotic critical value of the test *supF*(λ_1_, …, λ_*m*_, *q*) for significance level α.

Bai and Perron (23) extend the Chow test through sequential implementation and computation of the F-statistic for every possible structural break in the data. It tests the null hypothesis of *m* breakpoints versus the alternative of *m*+1 breakpoints using the sup-F statistics. In this case, the sup-F statistic is written as:


(10) $$\begin{array}{l} \text{supF}\left(m+1|m\right)\\ =\frac{\left[S_{T}\left({\hat{T}}_{1},\ldots ,{\hat{T}}_{m}\right)-\underset{1\leq i\leq m+1}{\max}\underset{\tau \in \Lambda_{i,\eta }}{\inf}S_{T}({\hat{T}}_{1},\ldots ,{\hat{T}}_{i-1},\;\ldots ,\;\tau ,{\hat{T}}_{i},\ldots ,{\hat{T}}_{m})\right]}{{\hat{\sigma }}^{2}} \end{array}$$

where


(11) $${\text{Λ}}_{i,\eta }=\left\{\tau ;{\hat{T}}_{i-1}+\left({\hat{T}}_{1},\ldots ,{\hat{T}}_{i-1}\right)\;\eta \leq \tau \leq {\hat{T}}_{i}-({\hat{T}}_{i},\ldots ,{\hat{T}}_{i-1})\eta \right\}$$

and where $S_{T}\left({\hat{T}}_{1},\ldots ,{\hat{T}}_{i-1},\;\ldots ,\;\tau ,{\hat{T}}_{i},\ldots ,{\hat{T}}_{m}\right)$ is the sum of squared residuals from least-squares estimation for each segment of the breaks, and ${\hat{\sigma }}^{2}$ is the variance estimator under null hypothesis. The procedure is conducted in sequence, beginning with testing the null hypothesis of no break vs alternative hypothesis of one break. When the null hypothesis is rejected, the first break is taken. Then a test for second break is conducted by testing the null hypothesis of one break vs alternative of two (1 + 1) breaks. The procedure continues in sequence until we fail to reject the null hypothesis. Carter and Smith (24) and Herrington and Tonsor (25) used these tests to assess structural change in grain markets and feedlot performance measures, respectively.

## Results

**Figure 3:**
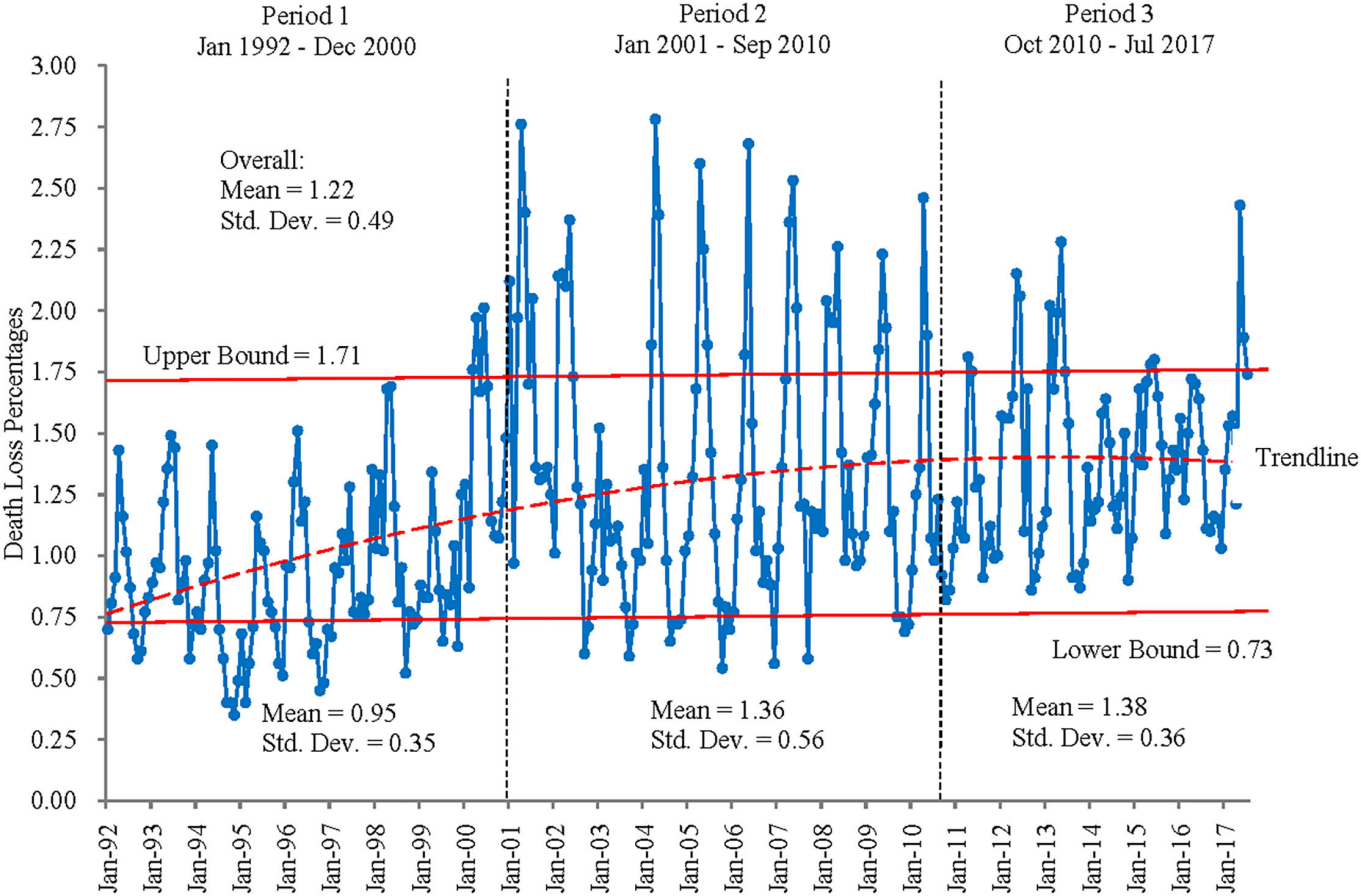
Death loss percentages for steers, Kansas, January 1992–July 2017.

**Figure 4:**
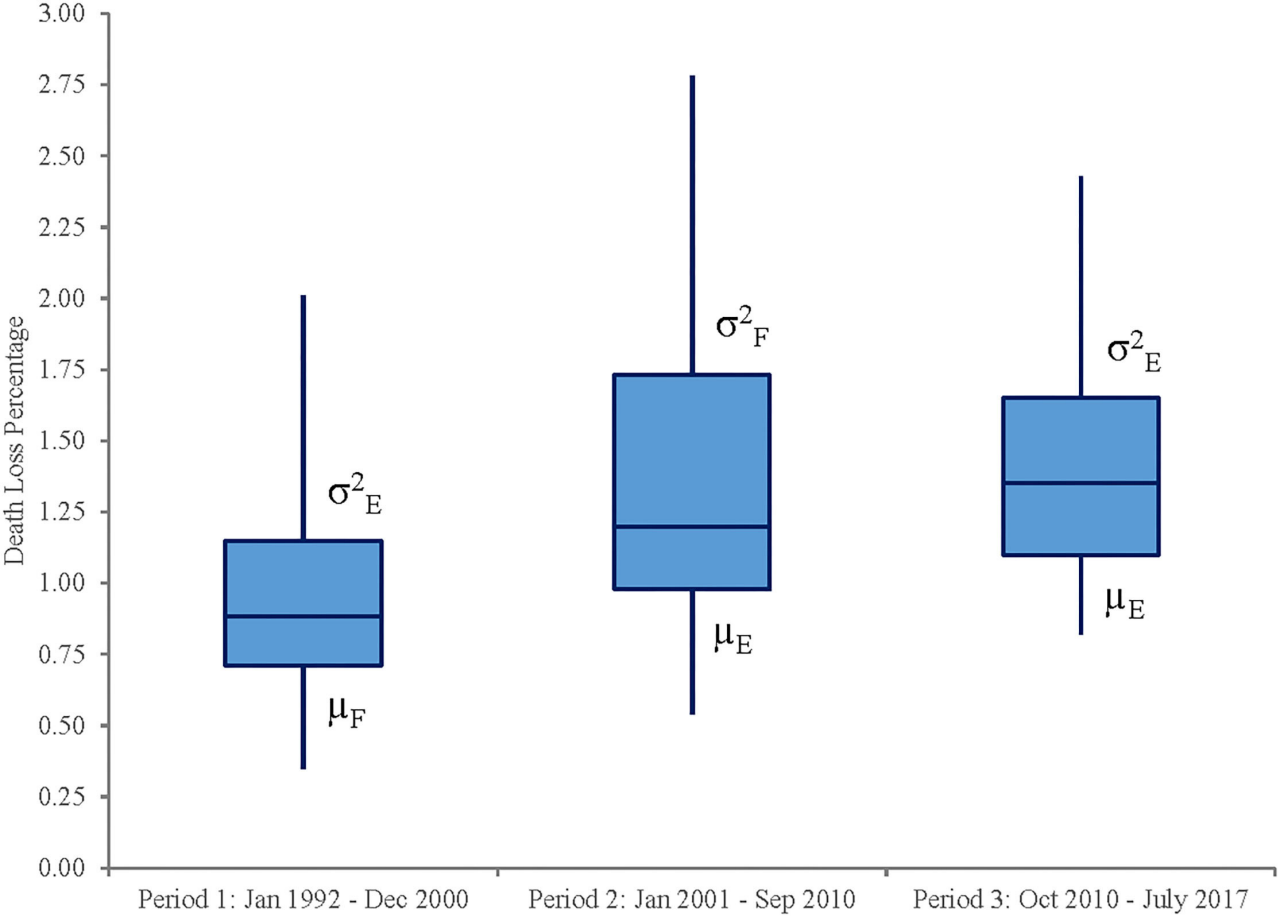
Box plot of death loss percentages for steers by periods.

Figure 3 illustrates monthly death loss percentages for steers in Kansas feedlots from January 1992 to July 2017. After visual inspection of magnitudes and variability across time, potential changes are examined by dividing the sample into period 1 (January 1992–December 2000), period 2 (January 2001–September 2010), and period 3 (October 2010–July 2017). Visually, these three periods do depict different magnitudes and variability of death loss rates over time, suggesting that structural change may have occurred across these periods. Results for tests of unequal means and variances across the three periods found statistical differences in these measures (Table 2). Period 1's mean is statistically different from the means in periods 2 and 3 (*p* = 0.00) and is significantly lower in magnitude. Though the mean death loss rates for periods 2 and 3 are not statistically different from each other (*p* = 0.713), their variances are statistically unequal (*p* = 0.00) as are the variances for periods 1 and 2 (*p* = 0.00). Box plots in Figure 4 illustrate these findings. Taken together, the results suggest that a shift (increase) occurred in both mean and variability of death loss rates between periods 1 and 2, but between periods 2 and 3, the mean death loss rate stayed the same while variability of death loss decreased significantly from period 2 to period 3.

**Table 2:** Results of tests for unequal means and variances of death loss percentages.

	**Period 1 and 2**	**Period 2 and 3**	**Period 1 and 3**	**Overall**
Variance difference	0.223	0.240	0.125	Pr > F
*p*-value for Pr > F	0.000	0.000	0.750	0.000
Mean difference	−0.405	−0.024	−0.429	Pr > F
*p*-value for Pr > |t|	0.000	0.713	0.000	0.000

### Initial model results

Model results for Equation 1 are presented in Table 3. All coefficients are positive, with the exception of the intercept and In-weight, which are negative but are not statistically significant. This is consistent with Babcock et al. (9) who found that in-weight influenced death loss in heifers, but had no significant impact on death loss in steer finishing. Most coefficients are significant at a 5% level including days on feed ($\hat{\beta_{2}}$), nine of the monthly dummies ($\hat{\delta_{k}}$), and time trend ($\hat{\beta_{3}}$) and *R*^2^ = 0.5053. Results indicate that days on feed has the highest impact on death loss percentage with higher death loss rates for longer feeding periods. The positive and statistically significant time trend coefficient demonstrates a gradual increase of death loss percentage over the sample period. Death loss percentage increased by 0.018% ($\hat{\beta_{3}}$ × mean of death loss percentage × 12 months) per year on average. Death loss for September closeouts (early fall) is significantly lower than other months. As is typical, late spring closeouts (April and May) have the highest death loss.

**Table 3:** Parameter estimates for Kansas feedlot death loss rate model (Equation 1), January 1992–July 2017.

**Variables**	**Parameter**	**Coefficients**	**Standard Error**
Intercept	$\hat{\beta_{0}}$	−5.1036	2.9380
Ln (In-Weight)	$\hat{\beta_{1}}$	−0.0514	0.6293
Ln (Days on feed)	$\hat{\beta_{2}}$	1.8934	0.3742^**^
Time trend	$\hat{\beta_{3}}$	0.0012	0.0004^**^
October	$\hat{\delta_{1}}$	0.0477	0.0489
November	$\hat{\delta_{2}}$	0.0873	0.0601
December	$\hat{\delta_{3}}$	0.1730	0.0635^**^
January	$\hat{\delta_{4}}$	0.2709	0.0656^**^
February	$\hat{\delta_{5}}$	0.2549	0.0677^**^
March	$\hat{\delta_{6}}$	0.3255	0.0721^**^
April	$\hat{\delta_{7}}$	0.4670	0.0835^**^
May	$\hat{\delta_{8}}$	0.4984	0.0842^**^
June	$\hat{\delta_{9}}$	0.3885	0.0764^**^
July	$\hat{\delta_{10}}$	0.2031	0.0648^**^
August	$\hat{\delta_{11}}$	0.1188	0.0510^**^
	ρ	0.4603	0.0508^**^
	R^2^	0.5053	

** Indicates significance at 5% level.

### Structural change

While inclusion of a time trend variable will capture some systematic change in death loss rates over time, tests for structural change can give more complete information about the nature of change and the need for modifications to the current model. The model estimated using Equation (1) was tested for indications of structural change using CUSUM, CUSUMSQ, and Bai and Perron testing procedures. The summary of breakpoints and close or coinciding events is shown in Table 4. The CUSUM detects instability in the intercept or systematic change in the model. Here, structural breaks are indicated at December 2000 and December 2001. Though the time trend already included in the model represents some systematic change in death loss over time, the breaks detected by CUSUM test indicate other yet unexplained factors.

**Table 4:** Structural change test results and coinciding events.

	**Test**	**Breakpoints indicated**	**Close or coinciding events**
Systematic	CUSUM	December 2000 December 2001	• Change of input combination in feed rations.
Abrupt	CUSUMSQ	December 2006	• Heavy snowstorms in the region (1000 mile path from central Oklahoma to northern Michigan) from November 30 to December 1. • Use of ractopamine hydrochloride (2004) and zilpaterol hydrochloride (mid-2007).
		May 2010 June 2010 September 2010	• Extreme heat during summer 2010.
Multiple Breakpoints	Bai and Perron	January 1996	• Abnormally cold and snowy conditions during winter 1995/96.
		December 2001	• Change of input combination in feed rations.

The CUSUMSQ test detects instability in the variance of the error terms, indicating the presence of abrupt change. This test detects breakpoints at December 2006, May 2010, June 2010, and September 2010.

Recall that Bai and Perron tests use the maximum F-statistic (sup-F) among F-statistics from all possible breakpoints or the maximum F statistic of Chow test. The Bai and Perron tests suggest two breakpoints; January 1996 and December 2001. The breakpoint of December 2001 detected through Bai and Perron tests aligns with breakpoints detected in CUSUM test (December 2000 and 2001).

The breakpoints detected by structural change tests implemented here are consistent with the test results for unequal means and variance (Figure 3 and Table 2). This led to modification of Equation 1 with the inclusion of a dummy variable for period 2 (December 2000–September 2010). This modified model is written as


(12) $$\begin{array}{l} \text{Ln}(DL_{t})=\beta_{0}+\beta_{1}\text{Ln}(INWT_{t})+\beta_{2}\text{Ln}(DOF_{t})+\beta_{3}t\\ \;+\sum_{k=1}^{11}\delta_{k}MD_{kt}+\gamma_{1}D1_{t}+\varepsilon_{t} \end{array}$$

where *D1* is a dummy for period 2 and other variables are previously defined in Equation 1.

**Figure 5:**
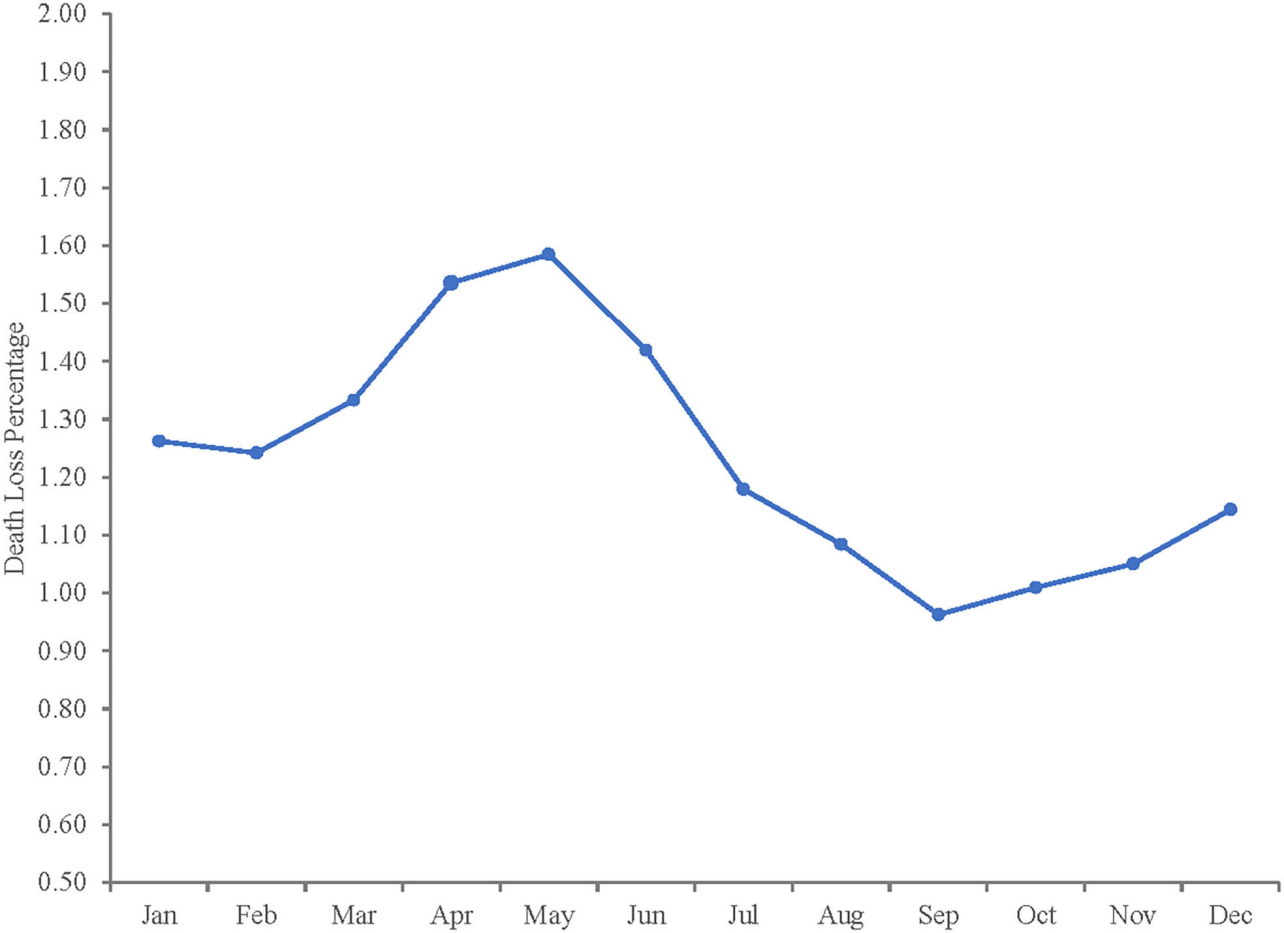
Seasonal pattern of death loss percentage from estimated model.

Table 5 reports parameter estimates from the modified model in Equation 11. As with the initial model, most are significant at the 5% level. The direction and magnitude of parameter estimates is also similar. When the structural change parameter for December 2000–September 2010 is included, in-weight still lacks significance and days on feed remains positive, statistically significant and is the largest influence on death loss rates. The seasonality component of death loss remains relatively stable, with September closeouts still exhibiting the lowest death loss rates and peaks in death loss rates for April and May closeouts. Figure 5 depicts the seasonal pattern of death loss estimated by the model. Time trend is still significant, indicating a gradual increase in death loss similar in magnitude to the basic model. The structural change dummy representing December 2000–September 2010 demonstrates that death loss is 0.117% ($\hat{\gamma_{1}}$ × mean of death loss percentage) higher on average during this period. For example, average monthly death loss rate is 1.22% for the whole sample period (January 1992–July 2017), resulting in an expected average monthly death loss rate during the period of December 2000–September 2010 of 1.337%. This result is consistent with the tests of unequal means and variances.

**Table 5:** Parameter estimates for Kansas feedlot death loss rate model with structural change parameter (Equation 11), January 1992–July 2017.

**Variables**	**Parameter**	**Coefficients**	**Standard error**
Intercept	$\hat{\beta_{0}}$	−6.4600	2.9565^**^
Ln(In-Weight)	$\hat{\beta_{1}}$	0.3095	0.6454
Ln(Days on feed)	$\hat{\beta_{2}}$	1.9363	0.3738^**^
Time trend	$\hat{\beta_{3}}$	0.0011	0.0004^**^
October	$\hat{\delta_{1}}$	0.0530	0.0486
November	$\hat{\delta_{2}}$	0.0927	0.0598
December	$\hat{\delta_{3}}$	0.1750	0.0632^**^
January	$\hat{\delta_{4}}$	0.2764	0.0654^**^
February	$\hat{\delta_{5}}$	0.2647	0.0676^**^
March	$\hat{\delta_{6}}$	0.3419	0.0722^**^
April	$\hat{\delta_{7}}$	0.4977	0.0843^**^
May	$\hat{\delta_{8}}$	0.5297	0.0851^**^
June	$\hat{\delta_{9}}$	0.4158	0.0770^**^
July	$\hat{\delta_{10}}$	0.2207	0.0649^**^
August	$\hat{\delta_{11}}$	0.1263	0.0507^**^
D1 (December 2000–September 2010)	$\hat{\gamma_{1}}$	0.0963	0.0466^**^
	ρ	0.4661	0.0507^**^
	R^2^	0.5103	

** Indicate significance at 5% level.

## Discussion

Industry data and anecdotal evidence suggest that death loss rates for feedlot cattle have increased over time. An important step toward greater understanding of changes in feedlot death loss is to understand the nature of the change at an aggregate level. Here, anecdotal evidence of change is supported by structural change tests and is reinforced by test of unequal means and variances conducted in this analysis. Catalysts of change may effect gradual, immediate or delayed impacts. Statistical evidence presented here suggests that systematic change does exist in the data and that a few events, such as extreme weather and feeding strategies, generated periods of abrupt change as well.

Producers may alter feed rations based on availability and relative prices of feed ingredients, which may be influenced indirectly by policy. While policy is not controllable by individual entities, producers make decisions based on the market environment created by the policy. For example, the Renewable Fuels Standard enacted in 2005 aims to reduce greenhouse gas emissions, but it also impacts corn prices – a primary input in cattle feeding. Increased prices and price risk associated with variation in corn prices might drive producers in cattle feeding operations to use alternative feed ingredients or feed additives with unclear long-term impacts. Increased demand for corn in ethanol processing meant less available for animal feed and also resulting in rising prices (26). This situation induced beef industry players to alter the input combination of feed rations by including the relatively less expensive by-product of the ethanol industry, distillers' grain.

Beta-agonists are one of several types of technology used in cattle feeding. The growth promotant ractopamine hydrochloride became commercially available in January 2004 as a feed additive designed to increase rate of gain and feed efficiency, thus potentially lowering feeding costs. In 2007, another beta-agonist feed additive, zilpaterol hydrochloride, was introduced to the cattle feeding industry as another option to enhance the natural ability of cattle to convert feed into lean meat. The use of zilpaterol hydrochloride was approved by U.S. Food and Drug Administration's Department of Health and Human Services in August 2006 and marketing commercially beginning in May 2007 (27).

The use of beta agonists has not been without controversy. Loneragan et al. (28) found that the use of beta agonists led to increased death loss rates when compared to rates for cattle where beta agonists were not used. Animal welfare concerns regarding the use of zilpaterol hydrochloride led Tyson Foods Inc. to ban zilpaterol hydrochloride-fed cattle from their beef operations in August 2013. Cargill, JBS, and National Beef Packing soon followed suit, representing over 80% of the beef packing industry. While zilpaterol hydrochloride was withdrawn from the market in 2013, ractopamine hydrochloride is still used and distillers' grain continues to be available from ethanol production plants. It is possible that the use of distillers' grain and zilpaterol hydrochloride in the post-Renewable Fuels Standard years impacted feedlot death loss (28, 29).

The distribution of feedlot cattle across different sources can impact feedlot death loss as well. Buda et al. (30) found that feedlot pens sourced directly from ranches had lower death loss rates than similar pens of sale barn cattle. The same study found that cattle sourced within the same geographical region as the feedlot location had lower death loss rates than pens sourced from other regions. While feedlots may have specific preferences for sourcing cattle, a host of things can impact availability of cattle from specific sources and may temporarily shift feedlot decisions regarding cattle source in spite of the accompanying increased death loss risk.

Uncontrollable factors such as weather may also impact feedlot death loss. Both severe cold and extreme heat often leads to increased death in feedlot cattle. From 1990 to 2016, there were multiple reports of abnormal weather conditions in the Southern Plains region. For example, there were early snowstorms in 1992 and 1997 and heavy snowstorms in winter 2006 (31). From fall 2010 through spring 2015, drought conditions in the Southern Plains region were considered extreme (32). Feedlot death loss may be higher than usual during such abnormal weather conditions, as such conditions place increased physical stress on cattle.

Recall that we divide the sample into period 1 (January 1992–December 2000), period 2 (January 2001–September 2010), and period 3 (October 2010–July 2017). Figures 3, 4 both indicate that the increased variance in death loss rates for period 2 is skewed toward more variability above rather than below the mean. Figure 3 shows that annual high death loss percentages for steers across the sample did not exceed the overall mean's upper bound (mean + standard deviation) of 1.71% before March 2000. However, it exceeded the upper bound often from March 2000 through the end of the second period in September 2010. The upper bound was exceeded less often in period 3, yet death loss never falls below the lower bound in period 3, resulting in a similar mean between periods 2 and 3 but a lower variance for period 3. The increased variability in death loss rates appears to coincide with the introduction of beta agonists in 2003, following a sustained period of rising corn prices. As feed rations began to include more fat and distillers' grains, supplements such as ractopamine hydrochloride (2003) and zilpaterol hydrochloride (2007) were used to improve cattle feeding efficiency. Interestingly, the return to more stability in death loss rates appears to coincide with the removal of zilpaterol hydrochloride from the market in 2013, though use of other beta agonists continues and the mean death loss rate remains higher relative to the pre-beta agonist period. It is possible that management related to ractopamine hydrochloride had improved over time such that variability in death loss related to its use decreased from its introduction to period 3.

In Table 4, the outcome of the three structural change tests tells a similar story regarding structural breaks. For example, the CUSUM test finds break points at December 2000 and December 2001, which also coincides with those feed ration changes, spurred by increasing corn prices, which may have influenced cattle health even prior to the introduction of beta agonists. Changes in implanting strategies for beef cattle production after USDA approval of the androgenic agent, trenbolone acetate, may also have contributed to structural change over the longer run (33, 34). The CUSUMSQ test finds breakpoints at December 2006, May 2010, June 2010, and September 2010. The closest event to December 2006 is heavy snowstorms in the region from November 30-December 1, 2006 (35). Hicks (31) reports that an estimated 10,000 to 30,000 head of feedlot cattle died in the Southern Great Plains due to these snowstorms. Similarly, for May, June, and September 2010, the coinciding event is extreme heat during summer 2010, as temperatures in the region for June and September were “much above average” and for July and August were “above average” (36). The coinciding event to the January 1996 structural break detected by Bai and Perron is the abnormal cold and snowy conditions during winter 1995/96 (37).

Overall, our analysis indicates a gradual increase in death loss rates over time, as well as a structural shift upward during the period of December 2000 – September 2010. Variance of death loss percentage is also higher during this period. Results also indicate that longer feeding periods lead to higher death loss rates. There may be ongoing factors that cause this systematic change, such as changes in feed ration and feeding technology, while beta agonist use and other events may have contributed to more abrupt changes. However, there is no clear evidence to directly associate these factors to feedlot death loss and such a study would require data at a more disaggregated level.

## Data availability statement

Publicly available datasets were analyzed in this study. This data can be found at: https://www.asi.k-state.edu/about/newsletters/focus-on-feedlots/monthly-reports.html.

## Author contributions

MB, KR, JR, and DP contributed to problem identification, model refinement, and paper concept. DP identified appropriate data. MB performed the regression analysis with direct input on modeling from KR. MB wrote the first draft. All authors contributed to manuscript revision and approved the submitted version.
